# The effect of acute high-intensity interval training and Tabata training on inhibitory control and cortical activation in young adults

**DOI:** 10.3389/fnins.2023.1229307

**Published:** 2023-09-14

**Authors:** Xueyun Shao, Longfei He, Yangyang Liu, Yang Fu

**Affiliations:** ^1^School of Sports, Shenzhen University, Shenzhen, China; ^2^Shenzhen Institute of Neuroscience, Shenzhen, China

**Keywords:** high-intensity interval training, Tabata training, inhibitory control, prefrontal cortex, functional near-infrared spectroscopy

## Abstract

**Introduction:**

Physical exercise not only benefits peoples’ health, but also improves their cognitive function. Although growing evidence suggests that high-intensity interval training (HIIT) is a time-efficient exercise regime that can improve inhibitory control performance by enhancing cortical activation in the prefrontal cortex, less is known about how Tabata training, a subset of HIIT that requires no equipment or facilities to perform, affects inhibitory control and cortical activation in young adults. Therefore, we aimed to reveal the effect of an acute bout of HIIT and Tabata training on inhibitory control and attempted to identify its potential neural substrates.

**Methods:**

Forty-two young adults (mean age: 19.36 ± 1.36 years; 21 females) performed the Stroop task and Simon task before and after acute HIIT, Tabata training, or a control session, and cortical hemodynamic changes in the prefrontal area were monitored by functional near-infrared spectroscopy (fNIRS) during the tasks. Both HIIT and Tabata interventions lasted for a total of 12 min. The HIIT participants performed ergometer cycling at their 80% maximal aerobic power at 90–100 rpm, and the Tabata participants performed a total of 8 intense activities, such as jumping jacks, high knees, and butt kickers, without using equipment or facilities, keeping the heart rate at 80–95% of their maximum heart rate. Participants in the control group watched a sport video while sedentary. Cognitive tasks data and fNIRS data were analyzed by repeated-measures three-way ANOVA.

**Results and discussion:**

Our results indicated that both the HIIT and Tabata groups exhibited reduced reaction times after the intervention, and there were alterations in activation patterns in the dorsolateral and ventrolateral prefrontal cortices.

## Introduction

Physical exercise is considered a common but effective way to improve physical and mental health across the life span (Ruegsegger and Booth, 2018; Schuch and Vancampfort, 2021).

While the majority of studies have investigated the acute effects of mild- or moderate-intensity exercise, recently, a growing number of researchers have focused on acute high-intensity interval training (HIIT). As mentioned in the World Health Organization guidelines, adults are recommended to regularly spend at least 150–300 min on moderate-intensity or 75–150 min on vigorous-intensity physical activity throughout each week to promote health benefits (World Health Organization, 2018). Lack of time has been acknowledged as one of the major barriers for people to maintain a physically active lifestyle (Rosenblat et al., 2020); considering this, there remains the need for an effective and time-efficient lifestyle intervention for adults. HIIT generally refers to the training protocol involving repeated sessions of short bursts of high-intensity exercise intercepted with periods of low-intensity recovery (Billat, 2001; Gibala and McGee, 2008). Many studies have indicated that HIIT can promote physiological and psychological benefits equal to or even greater than traditional moderate-intensity continuous aerobic training (Helgerud et al., 2007; Milanoviæ et al., 2015; Ramos et al., 2015). Therefore, HIIT has been proposed as a not only effective but also time-efficient alternative strategy for maintaining a physically active lifestyle.

There are various types of HIIT programs with different compositions and time spans, and Tabata training is one of them. Tabata training has been recognized as one of the most efficient forms of HIIT, because it can maximally improve both aerobic and anaerobic energy-supply systems (Tabata, 2019). With Tabata, there is standard 20 s work period followed by a 10 s rest period but with HIIT there is a recovery period which consists of some form of exercise (Tabata, 2019). Tabata was originally designed to train the elite athletes of the Japanese national speed skating team, but now it is used by the general public in scientific ways (Tabata et al., 1997). A Tabata training protocol is characterized by its unique training procedure, which comprises eight bouts of 20 s exercise followed by a 10 s rest (Tabata, 2019). HIIT requires the use of specialized laboratory equipment, typically performed on treadmills or cycle ergometers (Marriott et al., 2021). Compared with typical HIIT, Tabata is relatively inexpensive and usually requires very little equipment (Yan and Chen, 2022). For example, a modified version of Tabata, which is named the whole-body Tabata training protocol, does not necessitate the use of specialized equipment, thereby mitigating the need for access to facilities, which is more suitable for daily physical activity at home (McRae et al., 2012; Islam et al., 2020). Evidence has shown that this whole-body Tabata training protocol, including running and various body weight-bearing exercises (e.g., burpees and squat jumps), could maintain a high adherence rate (Chuensiri et al., 2018) and this evidence indicates that Tabata training is tolerable and positively accepted.

Inhibitory control, a core component of executive functions, refers to the ability to suppress irrelevant information and control prepotent responses as well as to resist interference from distracting stimuli. It is essential for the coordination of mental processes and action in accordance with current goals and plans and, ultimately, for successful living (Rey-Mermet et al., 2018; Tiego et al., 2018). Inhibitory control deficits have been implicated in clinical syndromes such as attention deficit hyperactivity disorder (Barkley, 1997), Tourette’s syndrome (Peterson et al., 1998), obsessive-compulsive disorders (Enright and Beech, 1993), and assorted “disinhibition syndromes” (Shulman, 1997). The prefrontal cortex (PFC) is a key structure in modulating high-level executive functions such as inhibitory control (Egner and Hirsch, 2005).

Inhibitory control can be assessed by the Stroop task and the Simon task (Stroop, 1935; Simon, 1969). In the traditional Stroop task, participants are presented with color words printed in different letter colors, and then they are required to name the color that the word is written in and ignore the word’s meaning (Cohen et al., 1990). In the traditional Simon task, participants are presented with a colored stimulus that appears on the left or the right of the fixation point; then they are required to press a button to report its color and ignore the spatial position of the stimulus (relative to the fixation point) (Craft and Simon, 1970; Proctor and Lu, 1994). Inhibitory control is necessary for participants to shift cognitive attention onto task-relevant information (typically based on the intrinsic characteristic of the stimulus, such as color), suppressing the automatic response to task-irrelevant information (typically based on an extrinsic characteristic of the stimulus, such as the word meaning or position) during the Stroop and Simon tasks (MacLeod, 1991; Proctor, 2011).

Cortical activation changes in the PFC can be assessed by a neuroimaging technique called functional near-infrared spectroscopy (fNIRS) (Kim et al., 2017; Herold et al., 2018). fNIRS is an optical and non-invasive method that monitors the cerebral hemodynamics of oxygenated and deoxygenated hemoglobin species (oxy-Hb and deoxy-Hb, respectively) by measuring alterations in the attenuation of near-infrared light passing through tissue (Koizumi et al., 2003; Obrig and Villringer, 2003). Unlike other neuroimaging methods, fNIRS is compact and portable. Moreover, fNIRS allows participants to perform cognitive tasks in a comfortable and natural environment instead of being confined to a small, restricted space, minimizing external disturbances.

Functional near-infrared spectroscopy studies have demonstrated that the PFC is the site of cortical activation after a single bout of exercise (Yanagisawa et al., 2010; Hyodo et al., 2012; Byun et al., 2014; Fujihara et al., 2021). Yanagisawa et al. (2010) found that an acute bout of moderate-intensity exercise increased the activation of the left dorsolateral prefrontal cortex (DLPFC) and improved cognitive performance of the Stroop task in young adults. Hyodo et al. (2012) indicated that the right frontopolar area (FPA) was activated by an acute bout of moderate-intensity exercise, which was associated with better Stroop performance in old adults. Byun et al. (2014) reported that 10 min of mild-intensity exercise enhanced the cortical activity of the left DLPFC and FPA with promotion of Stroop task performance compared to a control group of peers in young adults. Fujihara et al. (2021) compared the different effects of acute moderate-intensity exercise on PFC activity between young and older adults, finding a difference in the middle PFC activity between the two age groups: older adults showed an enhancement in the middle PFC activity after exercise, while there was no change in young adults.

Studies using fNIRS have revealed how acute HIIT affects inhibitory control, focusing on underlying neural substrates in adults (Kujach et al., 2018; Khandekar et al., 2022). Kujach et al. (2018) demonstrated that acute HIIT led to improved inhibitory control reflected by a shortening of the response time in the Stroop task, and it also stimulated cortical activation related to the Stroop task in the left DLPFC in young adults. Khandekar et al. (2022) showed that acute HIIT increased inhibitory control and that this improvement was associated with the activation of the PFC in young adults. Specifically, there was enhanced activation in the left DLPFC during the Stroop task and in the left FPA, left DLPFC, and right ventrolateral prefrontal cortex (VLPFC) during the Trail Making test (TMT) after the HIIT intervention.

Previous literature has presented evidence that an acute HIIT intervention improved inhibitory control and enhanced its associated hemodynamic activation in the PFC (Kujach et al., 2018; Khandekar et al., 2022). However, to our knowledge, changes in inhibitory control performance and its underlying cortical activation patterns after an acute Tabata intervention in young adults remain unclear and warrant further investigation. Thus, in this research, we adopted a whole-body Tabata training protocol and aimed to evaluate PFC activation through fNIRS response to acute HIIT or Tabata training during inhibitory control processing in young adults. Based on prior findings, we hypothesized that compared to the control condition, both acute HIIT and Tabata training would speed up reaction times and increase accuracy in inhibitory control tasks, and evoke PFC cortical activation in young adults, which is the neural substrate for acute cognitive improvement.

## Materials and methods

### Participants

Forty-two healthy, right-handed and normal weight, young adults voluntarily took part in this study. All the participants were college students recruited from Shenzhen University. The included participants were males (*n* = 21) and females (*n* = 21) aged between 18 and 22 years who had normal or corrected-to-normal vision with normal color vision. Participants were excluded if any of the following applied: (1) they answered Yes to any of the questions of the Physical Activity Readiness Questionnaire (PARQ), because if they did so, we would recommended they see a doctor before starting an exercise program; (2) they had a history of cardiorespiratory, cerebrovascular, or any disease limiting participants to exercise; (3) they had a history of neurological, major medical, or psychiatric disorders; (4) they had done any strenuous activity or consumed alcohol 24 h prior to the testing; and (5) they had taken medication at the time of measurement. Participant physical activity level during the preceding 7 days was evaluated using the International Physical Activity Questionnaire (IPAQ). None of the participants were excluded on the basis of these criteria. The demographic characteristics of the participants in the present study are listed in Table 1.

**TABLE 1:** Demographic characteristics of participants.

Variables	HIIT group (*n* = 14)	Tabata group (*n* = 15)	Control group (*n* = 13)	*p*-Value
Age (years)	19.07 ± 1.29	19.4 ± 1.35	19.62 ± 1.56	0.60
Sex (m/f)	6/8	8/7	7/6	0.82
Height (m)	1.67 ± 0.09	1.70 ± 0.09	1.67 ± 0.08	0.42
Weight (kg)	58.39 ± 7.68	62.60 ± 10.53	60.69 ± 9.72	0.49
BMI (kg/m^2^)	20.82 ± 2.05	21.32 ± 1.94	21.69 ± 2.61	0.59
HR_peak_ (bpm)	185.50 ± 7.70	185.33 ± 11.64	191.00 ± 6.57	0.11
MAP (watt)	135.36 ± 29.64	170.33 ± 53.40	159.23 ± 45.50	0.07

All values are presented as the mean (*M*) ± standard deviation (SD). BMI, body mass index; HR_peak_, peak heart rate; MAP, maximal aerobic power.

The participants were randomly subclassified into three groups: control (*n* = 13), HIIT (*n* = 14), and Tabata group (*n* = 15). We did simple randomization by using Excel. Every participant had an equal chance of participating in the control group and the experiment group. Before the experiment, all the participants were provided with informed consent forms and full information on the experimental protocol of the study. This study was approved by the ethical committee of the Medical School, Shenzhen University, Shenzhen, China (ethical code: PN-202200137 and PN-202300003).

### Experimental procedure

**FIGURE 1:**
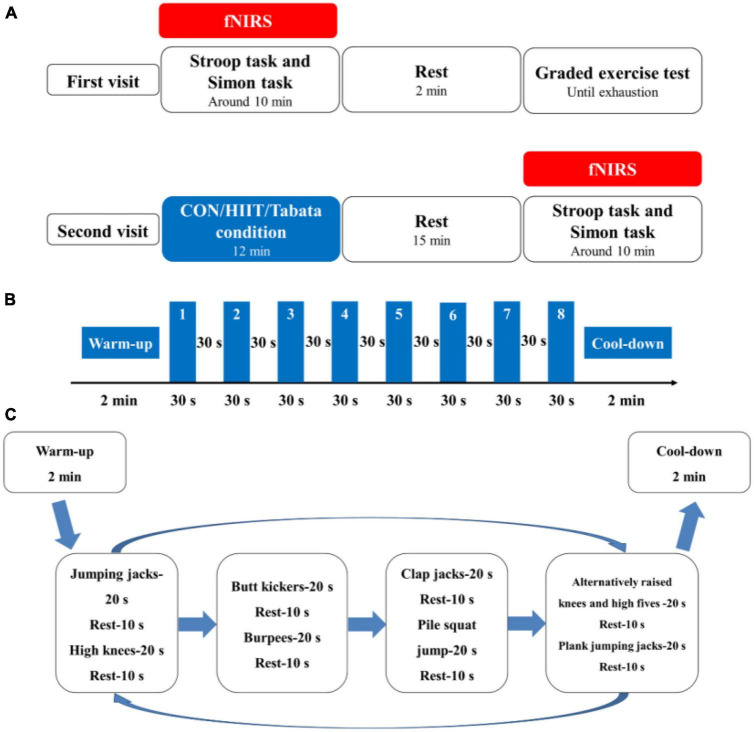
**(A)** Schematic illustration of the experimental procedures of the two visits for the control, HIIT, and Tabata groups. Using fNIRS, cortical hemodynamic activation was monitored while participants performed the Stroop task and Simon task. **(B)** Illustration of the acute HIIT protocol. **(C)** Illustration of the acute Tabata protocol.

Each participant was required to visit the laboratory two times, and the second visit was 48 h after the first visit (Figure 1). In their first visit, all the participants were randomly subclassified into three groups: the control, HIIT, and Tabata groups. They performed the Stroop task and Simon task at the beginning. Prefrontal hemodynamic changes were monitored by collecting fNIRS data during the Stroop task and Simon task. After participants completed the two cognitive tasks mentioned above, they had a 2 min rest, after which a graded exercise test was conducted using a cycle ergometer (Ergoselect 100, Ergoline GmbH, Baden-Württemberg, Germany) to determine individual maximal aerobic power (MAP) output. Specifically, participants began a warm-up cycling exercise for 3 min at 50 W, followed by a gradual increase in power demanded at a rate of 20 W (15 W for female participants) every 2 min (Table 2). The pedaling rate was kept between 55 and 60 rpm. Participants were verbally encouraged to achieve their maximum level. During the graded exercise test, each participant’s heart rate was monitored using a Shape heart rate monitor (Shenzhen Healthcare and Physical Education Data Technology Corporation, Shenzhen, China). The maximal heart rate (HR max) (beats/min) was determined by calculation using the formula: 220-age (in years). Each participant’s rate of self-perceived exertion (RPE) was measured by using the 10-point Borg Rating of Perceived Exertion scale (Borg, 1970). The HR and RPE were recorded every minute. We concluded the graded exercise test if participants had reached or exceeded 95% of their maximal heart rate (≥95% HR max), if participants’ RPE was 9 points or higher, if participants could not maintain the pedaling rate between 55 and 60 rpm although we kept verbally encouraging them, or if participants voluntarily announced they were so exhausted to preserve this test. When a participant competed the graded exercise test, the final power of the cycle ergometer was considered as the participant’s own MAP.

**TABLE 2:** Graded exercise test protocol.

Stage	Time period (min)	Duration (min)	Power (W)		Pedaling rate (rpm)	Type of exercise
			Male	Female		
1	0–3	3	50	50	60	Cycling (warm-up)
2	3–5	2	70	65	55–60	Cycling
3	5–7	2	90	80	55–60	Cycling
4	7–9	2	110	95	55–60	Cycling
5	9–11	2	130	115	55–60	Cycling
6	11–13	2	150	130	55–60	Cycling
…						
Until exhaustion						

In the second visit, both the HIIT and the Tabata intervention lasted for a total of 12 min. At the beginning of the HIIT intervention, participants performed a warm-up for 2 min at 50 W and at 55 to 60 rpm; then they performed an HIIT session of 8 minutes. Afterwards, they performed a cool-down for 2 min at 50 W and at 55 to 60 rpm on a cycle ergometer. The 8 min HIIT intervention consisted of eight repetitions of 30 s of ergometer cycling at 80% MAP at 90 to 100 rpm and 30 s recovery at 40% MAP at 55 to 60 rpm. We adopted and modified a whole-body Tabata training protocol based on previous research (McRae et al., 2012; Islam et al., 2020). At the beginning of our whole-body Tabata intervention, participants performed a warm-up consisting of overhead arm reaches, arm circles and shoulder shrugs, hip rotations and hip circles, lunges, and crossover toe touches for a total of 2 min, and then they performed an 8 min Tabata intervention without using equipment or facilities, after which they performed a cool-down stretching for 2 min. The 8 min Tabata intervention consisted of two repeated circuits of Tabata exercise, and each circuit lasted for 4 min, broken up into eight 30 s sets. Each 30 s period was broken into 20 s of workout and 10 s of rest. Specifically, in each circuit, participants performed a total of 8 intense activities: jumping jacks, high knees, butt kickers, burpees, clap jacks, plie squat jump, alternatively raised knees and high fives, and plank jumping jacks sequentially. During the Tabata intervention, participants wore the Shape heart rate monitor and were required to reach 80% to 95% of their own HR max based on the American College of Sports Medicine (ACSM) guidelines (Garber et al., 2011). For safety reasons, we did not elevate participants’ heart rate to a near-maximal level. In the control group, participants watched a sports documentary, sitting sedentary for 10 min, after which they had a 2 min rest. Participants in the HIIT and Tabata groups completed their interventions and rested for 15 min, and all the participants performed the two cognitive tasks again monitored by fNIRS.

### Behavioral measurements

**FIGURE 2:**
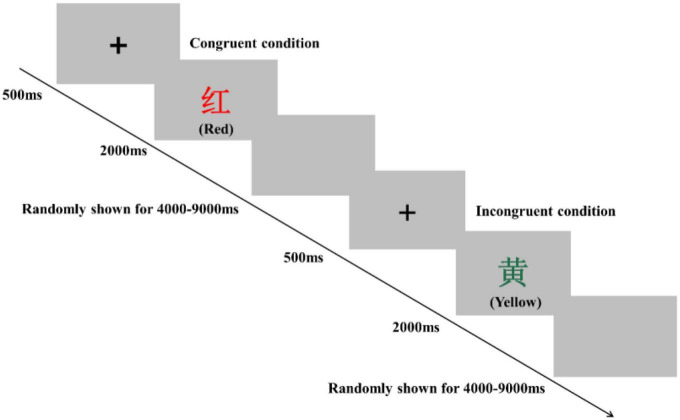
Schematic illustration of the Stroop task. Examples of single trials for the congruent and incongruent conditions are illustrated. The presented words were written in Chinese. Their translations into English are denoted in parentheses.

Inhibitory control performance was assessed by using a modified version of the Stroop task and Simon task in an event-related design. The order of these two tests was counterbalanced pre- and postsessions in all three groups to eliminate bias. These two tasks were programmed and presented with E-Prime 3.0 software (Psychology Software Tools Inc., United States). The participants sat approximately 80 cm in front of a 15-inch LCD screen with a gray background and responded to the task using the computer keyboard. In the Stroop task, participants were instructed to determine the color of the word. Specifically, they were required to respond with a left-hand press (“D” key) when the current color of the word was red, with a left-hand press (“F” key) when the current color of the word was yellow, with a right-hand press (“J” key) when the current color of the word was blue, and with a right-hand press (“K” key) when the current color of the word was green. The Stroop task consisted of 80 trials, including 40 congruent conditions and 40 incongruent conditions, presented in random order (Figure 2). For the congruent condition, the color words “RED,” “YELLOW,” “BLUE,” or “GREEN” were printed in the congruent color (e.g., “RED” word was printed in red color). For the incongruent condition, the color words “RED,” “YELLOW,” “BLUE,” or “GREEN” were printed in an incongruent color (e.g., “YELLOW” word was printed in blue color) to produce cognitive interference between the color word and the color. At the beginning of each trial, a fixation cross was presented 500 ms on the center of the screen to catch participants’ attention; then, the word stimulus was presented on the center of the computer screen. The word stimulus remained on the screen until the response was given by participants or for 2,000 ms. Each trial was separately presented with an interstimulus interval randomly showing a blank screen for 4,000–9,000 ms to avoid prediction of the timing of the subsequent trial. The Stroop words displayed on the LCD screen were all presented in Chinese. Before the start of the formal experiment, a practice session consisting of 12 trials was performed.

**FIGURE 3:**
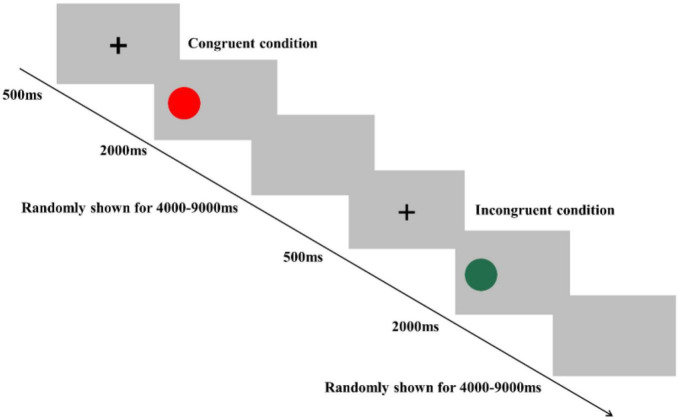
Schematic illustration of the Simon task. Examples of single trials for congruent and incongruent conditions are illustrated.

In the Simon task, participants were instructed to determine the color of the stimulus. Specifically, they were asked to press a left-hand key (“F” key) when the color of the stimulus was red and press a right-hand key (“J” key) when the color of the stimulus was green. The Simon task consisted of 80 trials, including 40 congruent conditions and 40 incongruent conditions, presented in random order (Figure 3). In the congruent condition, the stimulus was presented on the location (relative to fixation) that matched the required key-press (e.g., the red colored stimulus appeared on the left side of fixation). In the incongruent condition, the stimulus was presented in the opposite location that conflicted with the required key press (e.g., the red colored stimulus appeared on the right side of fixation) to produce cognitive interference between the spatial location of the stimulus and the appropriate key press response. At the beginning of each trial, a fixation cross was presented for 500 ms at the center of the screen to catch participants’ attention; then, the stimulus was presented on the left or right side of the computer screen. The stimulus remained on the screen until the response was given by participants, or for 2,000 ms. Each trial was separately presented with an interstimulus interval randomly showing a blank screen for 4,000–9,000 ms to avoid prediction of the timing of the subsequent trial. Before starting the formal experiment, a practice session consisting of eight trials was performed. Reaction time (RT) and accuracy rate (ACC) were recorded in both the Stroop and Simon tasks.

### fNIRS measurements

We used the portable Brite Artinis fNIRS version 24 Brain imaging system (Artinis Medical Systems, Netherlands), applying 2 wavelengths of near-infrared light (760 and 850 nm) to monitor cortical hemodynamic changes. Optical density data were analyzed using the modified Beer-Lambert Law to calculate signals reflecting oxy-Hb, deoxy-Hb, and total hemoglobin (total Hb) signal changes in millimolar-millimeter (mM**⋅**mm) units (Maki et al., 1995). Compared to deoxy-Hb and total-Hb signals, oxy-Hb signals have a higher signal-to-noise ratio (Strangman et al., 2002) and retest reliability (Plichta et al., 2006). Therefore, this study used oxy-Hb signals as indicators of regional cortical activation. The sampling rate was set at 25 Hz.

**FIGURE 4:**
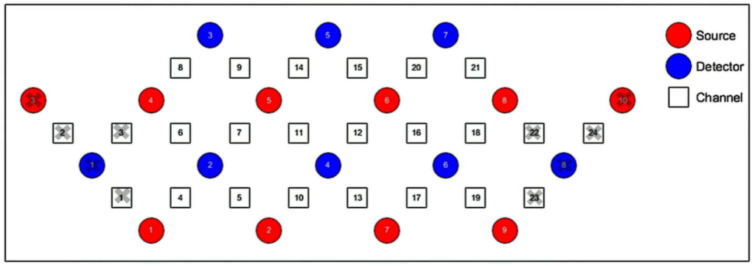
Schematic representation of the arrangement of 24 fNIRS channels of the PFC on a 2D view. The illuminating probes (source) and detecting probes (detector) are printed with red and blue colors, respectively.

We used 1 set of Brite Frontal 24-channel optode templates, consisting of 10 illuminating and 8 detecting probes arranged alternately at an interprobe distance of 3 cm, resulting in 24 channels (CH) that were bilaterally symmetrically distributed (Figure 4). Among the 24 channels, we excluded those located outside the PFC (channels 1, 2, 3, 22, 23, and 24). The probability of estimating spatial information for each channel of a brain region was determined using the probabilistic estimation method (Okamoto et al., 2004; Tsuzuki et al., 2007). The optodes were placed on the head of the participants according to the topographic probe layout map along with the international standard EEG 10-20 coordinate system (Homan et al., 1987; Herwig et al., 2003), and channel grids were placed to cover the following regions of interest (ROIs) for each hemisphere: DLPFC, VLPFC, and FPA over the PFC.

We employed virtual registration (Tsuzuki et al., 2007) to register fNIRS data to Montreal Neurological Institute (MNI) standard brain space (Brett et al., 2002). We performed a statistical analysis of the MNI coordinate values for the fNIRS channels to obtain the most likely estimate of the location of given channels for the group subjects, and spatial variability associated with the estimation (Singh et al., 2005). Finally, we anatomically labeled the estimated locations using a MATLAB function that reads anatomical labeling information coded in a macroanatomical brain atlas (Shattuck et al., 2008).

### fNIRS data analysis

For raw fNIRS data, preprocessing was conducted using the open-source software HOMER2 implemented in MATLAB (MathWorks). All recorded signals were first converted to optical density for processing in the HOMER2 software. Channels with low amplitude were excluded from group processing. Correction for motion artifacts was performed using wavelet filtering, which has been described as a promising approach to reduce the influence of motion artifacts (Brigadoi et al., 2014; Jahani et al., 2018). After this processing, the signals were then bandpass filtered with frequencies between 0.01 and 0.3 Hz to remove baseline drift and physiological noise. Finally, these filtered signals were converted to oxy-Hb concentrations. We obtained channelwise and subjectwise contrasts from the preprocessed time-series data by calculating the intertrial mean of differences between the oxy-Hb signals of peak (5–10 s after the onset on trial) and baseline (0–2 s before the onset of trial) periods. The contrasts obtained were subjected to a second level of random effects group analysis.

**FIGURE 5:**
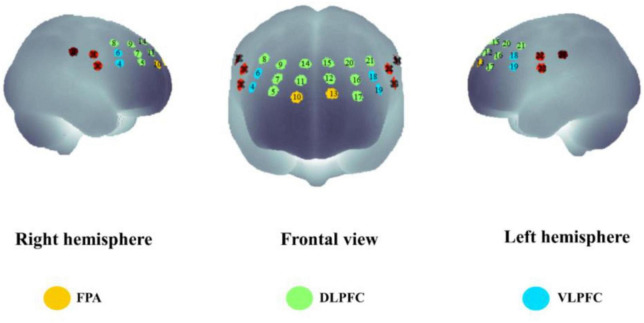
The spatial distribution of all the fNIRS channels used in the present study. Channel numbers are denoted above the corresponding location. Three lateral subregions of the prefrontal cortex are printed with colors [yellow, frontopolar area (FPA); green, dorsolateral prefrontal cortex (DLPFC); blue, ventrolateral prefrontal cortex (VLPFC)].

We performed statistical analyses on ROIs, and we classified activated channels into six ROIs throughout the PFC, including the left DLPFC (L-DLPFC; channels 12, 15, 16, 17, 20, and 21), the left VLPFC (L-VLPFC; channels 18 and 19), the left FPA (L-FPA; channel 13), the right DLPFC (R-DLPFC; channels 5, 7, 8, 9, 11, and 14), the right VLPFC (R-VLPFC; channels 4 and 6), and the right FPA (R-FPA; channel 10) (Figure 5).

### Statistical analysis

Statistical analyses were performed using SPSS Statistics version 24 (IBM Corp., Chicago, IL, USA). First, we determined the normality of the cognitive data by using the Shapiro–Wilk test. Then, the RT and ACC of the Stroop task and Simon task were subjected to repeated-measures three-way ANOVA with group (control/HIIT/Tabata) the as between-subject factor and with condition (congruent/incongruent) and time (pre/post) as within-subject factors. Partial eta-squared was used to report the effect size for the significant main and interaction effect. All data are presented as the mean ± SD. Statistical significance levels were set at a *p*-value of <0.05. The Bonferroni method was adopted to control for controlling familywise errors.

After preprocessing the raw signals, we converted fNIRS data to ROI-wise data on both sides of the PFC. The data were then subjected to repeated-measures three-way ANOVA with group (control/HIIT/Tabata) as the between-subject factor and with condition (congruent/incongruent) and time (pre/post) as the within-subject factors. Statistical significance levels were set at a *p*-value of <0.05. The Bonferroni method was adopted to control for familywise errors.

## Results

### Physiological aspects of HIIT and Tabata intervention

We monitored HR and RPE every minute during exercise to investigate the physiological characteristics of the acute HIIT and Tabata intervention. The mean value ± SD of HR and RPE during the HIIT and Tabata interventions were 168.89 ± 9.33 beats/min, 7.79 ± 1.96 points, and 168.78 ± 10.38 beats/min, 8.00 ± 1.79 points, respectively. These HR and RPE parameters showed that the exercise intensity of HIIT and Tabata intervention was vigorous according to the classification of the ACSM guidelines (Garber et al., 2011).

### Behavioral results

#### Stroop task

The RT and ACC of the Stroop task before and after the intervention in the control, HIIT, and Tabata groups are shown in Table 3. The ANOVA for RT and ACC showed that there was no significant condition × time × group interaction for both RT and ACC in the Stroop task (*F*(2,82) = 0.762, *p* = 0.474, partial η^2^ = 0.038, Bonferroni-corrected; *F*(2,82) = 2.104, *p* = 0.136, partial η^2^ = 0.097, Bonferroni-corrected, respectively). For RT, there was a significant main effect of the condition [*F*(2,82) = 154.514, *p* < 0.001, partial η^2^ = 0.798, incongruent > congruent] and time [*F*(2,82) = 22.464, *p* < 0.001, partial η^2^ = 0.365, pre > post]. For ACC, there was a significant main effect of the condition [*F*(2,82) = 17.009, *p* < 0.001, partial η^2^ = 0.304, congruent > incongruent]. The main effect of condition verified that the Stroop effect could be generally observed in this study.

**TABLE 3:** Reaction time and ACC in the Stroop task in the control, HIIT, and Tabata groups over the acute intervention.

Variables	Control group				HIIT group				Tabata group			
	Pre		Post		Pre		Post		Pre		Post	
	Congruent	Incongruent	Congruent	Incongruent	Congruent	Incongruent	Congruent	Incongruent	Congruent	Incongruent	Congruent	Incongruent
RT (ms)	651.43 ± 100.26	774.03 ± 130.33	618.08 ± 95.55	723.76 ± 153.61	660.22 ± 116.01	803.64 ± 175.95	588.89 ± 102.50	685.91 ± 147.31	759.68 ± 167.28	909.77 ± 167.30	694.87 ± 147.78	811.73 ± 161.61
ACC (%)	96.73 ± 3.13	95.77 ± 2.14	95.77 ± 5.24	94.04 ± 7.18	98.04 ± 2.12	96.96 ± 2.80	98.21 ± 2.06	95.54 ± 3.28	97.50 ± 3.41	94.67 ± 5.16	98.33 ± 2.44	97.33 ± 2.58

Data are presented as the mean ± SD. RT, reaction time; ACC, accuracy.

#### Simon task

The RT and ACC of the Simon task before and after the intervention in the control, HIIT, and Tabata groups are shown in Table 4. The ANOVA for RT and ACC showed that there was no significant condition × time × group interaction for both RT and ACC in the Simon task [*F*(2,82) = 1.503, *p* = 0.235, partial η^2^ = 0.072, Bonferroni-corrected; *F*(2,82) = 0.837, *p* = 0.441, partial η^2^ = 0.041, Bonferroni-corrected, respectively]. For RT, there was a significant main effect of the condition [*F*(2,82) = 13.620, *p* < 0.001, partial η^2^ = 0.259, incongruent > congruent]. For ACC, there was no significant main effect. The main effect of condition indicated that the Simon effect was generally observed in this study.

**TABLE 4:** Reaction time and ACC of the Simon task in the control, HIIT, and Tabata groups after the acute intervention.

Variables	Control group				HIIT group				Tabata group			
	Pre		Post		Pre		Post		Pre		Post	
	Congruent	Incongruent	Congruent	Incongruent	Congruent	Incongruent	Congruent	Incongruent	Congruent	Incongruent	Congruent	Incongruent
RT (ms)	451.42 ± 49.05	478.95 ± 47.79	477.79 ± 104.08	488.16 ± 101.08	460.45 ± 78.41	473.06 ± 73.91	418.87 ± 73.42	431.83 ± 70.73	514.65 ± 96.48	539.98 ± 121.16	507.13 ± 98.86	508.81 ± 95.52
ACC (%)	98.46 ± 1.92	97.50 ± 3.31	98.27 ± 2.58	97.12 ± 3.51	98.21 ± 2.28	98.57 ± 1.89	97.86 ± 1.66	97.86 ± 1.93	95.83 ± 6.17	95.00 ± 7.07	97.83 ± 3.76	98.17 ± 3.06

Data are presented as the mean ± SD. RT, reaction time; ACC, accuracy.

### fNIRS results

#### Stroop task

**FIGURE 6:**
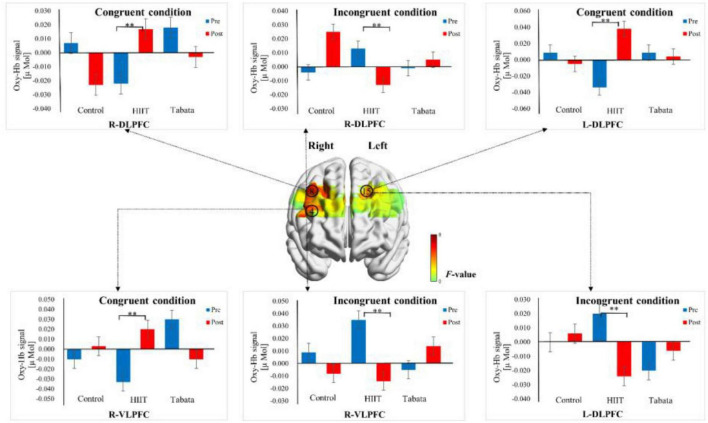
Cortical activation patterns during the Stroop task in the control, HIIT, and Tabata groups. F-map of the oxy-Hb signal showing the significant condition × time × group interaction. Among the six regions of interest, a significant interaction can be seen in the R-DLPFC (channel 8), R-VLPFC (channel 4), and L-DLPFC (channel 15) (*p* < 0.05, Bonferroni-corrected). *F*-values are shown according to the color bar. ^**^Means there was a significant difference of cortical activation over time.

Changes in the oxy-Hb concentrations in the Stroop task before and after intervention in the control, HIIT, and Tabata groups are presented in Table 5. The ANOVA for the six ROIs on oxy-Hb signals revealed that there was a significant condition × time × group interaction in the R-DLPFC [*F*(2,82) = 5.496, *p* = 0.011, partial η^2^ = 0.323, Bonferroni-corrected, channel 8], R-VLPFC [*F*(2,82) = 5.793, *p* = 0.013, partial η^2^ = 0.420, Bonferroni-corrected, channel 4], and L-DLPFC [*F*(2,82) = 5.142, *p* = 0.023, partial η^2^ = 0.442, Bonferroni-corrected, channel 15] (Figure 6). Specifically, in all R-DLPFC, R-VLPFC, and L-DLPFC regions, the participants in the HIIT group showed significantly increased oxy-Hb signals over time while they performed the congruent condition trials of the Stroop task [*F*(2,82) = 6.775, *p* = 0.016, R-DLPFC; *F*(2,82) = 5.414, *p* = 0.033, R-VLPFC; *F*(2,82) = 17.297, *p* = 0.001, L-DLPFC], and participants in the HIIT group showed significantly decreased oxy-Hb signals over time while they performed the incongruent condition trials of the Stoop task [*F*(2,82) = 6.775, *p* = 0.016, R-DLPFC; *F*(2,82) = 9.863, *p* = 0.006, R-VLPFC; *F*(2,82) = 4.857, *p* = 0.046, L-DLPFC]. These results demonstrated that an acute bout of HIIT intervention stimulated congruent-related cortical activation and diminished incongruent-related cortical activation in the R-DLPFC, R-VLPFC, and L-DLPFC. However, there were no significant oxy-Hb hemodynamic changes in participants in the control or Tabata group over time when they were performing different condition trials.

**TABLE 5:** Hemodynamic concentrations in the Stroop task in all channels.

		Control group (μ mol)		HIIT group (μmol)		Tabata group (μ mol)	
Channels	Condition	Pre	Post	Pre	Post	Pre	Post
4	Congruent	**−0.015 ± 0.04**	**0.003 ± 0.01**	**−0.038 ± 0.03**	**0.00001 ± 0.06**	**0.030 ± 0.03**	**−0.006 ± 0.04**
	Incongruent	**0.014 ± 0.02**	**−0.008 ± 0.02**	**0.040 ± 0.03**	**−0.005 ± 0.04**	**−0.005 ± 0.04**	**0.013 ± 0.02**
5	Congruent	−0.0005 ± 0.04	0.006 ± 0.03	0.005 ± 0.04	0.028 ± 0.05	0.025 ± 0.03	−0.011 ± 0.05
	Incongruent	0.006 ± 0.03	−0.010 ± 0.02	0.004 ± 0.04	−0.014 ± 0.06	−0.005 ± 0.03	−0.014 ± 0.05
6	Congruent	0.008 ± 0.03	−0.003 ± 0.02	0.004 ± 0.04	0.016 ± 0.04	0.023 ± 0.05	−0.002 ± 0.04
	Incongruent	0.084 ± 0.29	0.002 ± 0.02	−0.002 ± 0.04	−0.011 ± 0.04	−0.002 ± 0.06	−0.011 ± 0.05
7	Congruent	0.028 ± 0.12	0.001 ± 0.05	−0.005 ± 0.02	0.029 ± 0.06	0.004 ± 0.04	−0.007 ± 0.04
	Incongruent	0.204 ± 0.62	−0.026 ± 0.04	−0.002 ± 0.03	−0.033 ± 0.07	0.003 ± 0.02	−0.010 ± 0.03
8	Congruent	**−0.0002 ± 0.03**	**−0.018 ± 0.04**	**−0.019 ± 0.03**	**0.024 ± 0.04**	**0.016 ± 0.03**	**−0.006 ± 0.03**
	Incongruent	**0.302 ± 0.91**	**0.021 ± 0.04**	**0.010 ± 0.03**	**−0.027 ± 0.09**	**−0.002 ± 0.03**	**−0.002 ± 0.04**
9	Congruent	−0.074 ± 0.24	0.006 ± 0.03	−0.026 ± 0.05	0.005 ± 0.10	−0.020 ± 0.08	0.028 ± 0.08
	Incongruent	0.507 ± 1.23	0.009 ± 0.03	0.014 ± 0.05	0.003 ± 0.08	0.040 ± 0.11	0.019 ± 0.03
10	Congruent	−0.011 ± 0.04	0.013 ± 0.03	−0.002 ± 0.04	0.013 ± 0.06	0.026 ± 0.03	0.016 ± 0.02
	Incongruent	0.429 ± 1.39	−0.010 ± 0.04	−0.006 ± 0.04	−0.011 ± 0.06	−0.017 ± 0.03	−0.019 ± 0.04
11	Congruent	0.022 ± 0.10	−0.0001 ± 0.04	−0.015 ± 0.03	0.023 ± 0.04	0.011 ± 0.04	0.011 ± 0.02
	Incongruent	0.763 ± 2.00	−0.003 ± 0.03	0.011 ± 0.04	−0.013 ± 0.05	0.007 ± 0.05	−0.015 ± 0.04
12	Congruent	0.050 ± 0.15	−0.008 ± 0.03	−0.003 ± 0.03	0.017 ± 0.04	0.010 ± 0.03	0.006 ± 0.02
	Incongruent	0.852 ± 2.34	0.012 ± 0.03	−0.010 ± 0.04	−0.009 ± 0.04	−0.005 ± 0.04	−0.002 ± 0.04
13	Congruent	−0.002 ± 0.03	−0.003 ± 0.03	−0.0001 ± 0.03	0.015 ± 0.03	0.020 ± 0.04	0.004 ± 0.04
	Incongruent	0.751 ± 2.31	0.011 ± 0.04	−0.002 ± 0.03	−0.016 ± 0.04	−0.010 ± 0.03	−0.017 ± 0.05
14	Congruent	0.061 ± 0.12	−0.002 ± 0.02	−0.017 ± 0.03	0.019 ± 0.04	−0.012 ± 0.03	0.009 ± 0.02
	Incongruent	−0.024 ± 0.04	0.001 ± 0.02	0.007 ± 0.04	−0.007 ± 0.04	0.006 ± 0.03	−0.018 ± 0.06
15	**Congruent**	**0.034 ± 0.09**	**0.001 ± 0.02**	**−0.011 ± 0.05**	**0.029 ± 0.05**	**0.007 ± 0.03**	**0.006 ± 0.02**
	**Incongruent**	**1.256 ± 3.32**	**0.008 ± 0.02**	**−0.005 ± 0.06**	**−0.013 ± 0.06**	**−0.010 ± 0.05**	**−0.026 ± 0.09**
16	Congruent	0.007 ± 0.11	0.010 ± 0.03	−0.004 ± 0.04	0.025 ± 0.07	0.010 ± 0.04	0.004 ± 0.01
	Incongruent	1.108 ± 3.31	0.007 ± 0.04	−0.001 ± 0.05	−0.011 ± 0.06	0.0001 ± 0.04	−0.024 ± 0.05
17	Congruent	−0.002 ± 0.03	−0.012 ± 0.05	−0.013 ± 0.04	0.001 ± 0.06	0.013 ± 0.05	0.002 ± 0.04
	Incongruent	1.028 ± 3.32	0.017 ± 0.03	0.016 ± 0.04	0.001 ± 0.05	−0.001 ± 0.03	−0.022 ± 0.05
18	Congruent	0.001 ± 0.04	0.007 ± 0.03	−0.015 ± 0.02	0.029 ± 0.05	0.015 ± 0.05	0.003 ± 0.05
	Incongruent	1.087 ± 3.61	0.002 ± 0.03	0.010 ± 0.04	−0.011 ± 0.05	−0.001 ± 0.04	−0.014 ± 0.06
19	Congruent	−0.004 ± 0.03	−0.007 ± 0.03	−0.025 ± 0.03	0.016 ± 0.04	−0.013 ± 0.12	0.012 ± 0.06
	Incongruent	0.009 ± 0.02	0.018 ± 0.03	0.024 ± 0.04	−0.010 ± 0.04	0.008 ± 0.05	−0.021 ± 0.06
20	Congruent	−0.007 ± 0.04	0.004 ± 0.03	−0.028 ± 0.07	0.050 ± 0.08	−0.016 ± 0.13	0.018 ± 0.07
	Incongruent	0.004 ± 0.03	0.018 ± 0.03	0.019 ± 0.06	−0.025 ± 0.06	0.039 ± 0.13	0.037 ± 0.04
21	Congruent	−0.006 ± 0.03	0.012 ± 0.03	−0.009 ± 0.02	0.025 ± 0.04	0.010 ± 0.03	0.002 ± 0.04
	Incongruent	0.006 ± 0.03	−0.001 ± 0.02	0.001 ± 0.04	0.007 ± 0.05	0.001 ± 0.02	−0.011 ± 0.06

Oxy-Hb concentrations (μmol) from pre- to post-test in the control, HIIT, and Tabata groups are presented. Data are presented as the mean ± SD. Bold values indicate a significant condition × time × group interaction (*p* < 0.05).

#### Simon task

**FIGURE 7:**
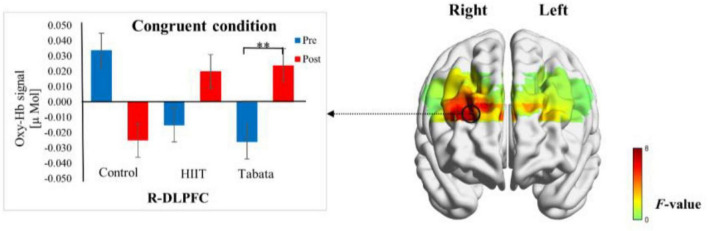
Cortical activation patterns during the Simon task in the control, HIIT, and Tabata groups. F-map of the oxy-Hb signal showing the significant condition × time × group interaction. Among the six regions of interest, a significant interaction can be seen in the R-DLPFC (channel 5) (*p* < 0.05, Bonferroni-corrected). *F*-values are shown according to the color bar. ^**^Means there was a significant difference of cortical activation over time.

Changes in the oxy-Hb concentrations in the Simon task before and after intervention in the control, HIIT, and Tabata groups are presented in Table 6. The ANOVA for the six ROIs on oxy-Hb signals revealed that there was a significant condition × time × group interaction in the R-DLPFC [*F*(2,82) = 6.275, *p* = 0.005, partial η^2^ = 0.295, Bonferroni-corrected, channel 5] (Figure 7). Specifically, in the R-DLPFC regions, the participants in the Tabata group showed significantly increased oxy-Hb signals over time while they performed the congruent condition trials of the Simon task [*F*(2,82) = 6.843, *p* = 0.014], but there were no significant differences in oxy-Hb signals while they performed the incongruent condition trials of the Simon task [*F*(2,82) = 0.004, *p* = 0.948]. These results demonstrated that an acute bout of Tabata intervention stimulated congruent-related cortical activation in the R-DLPFC. However, there were no significant oxy-Hb hemodynamic changes among participants in the control or HIIT group over time when they were performing different condition trials.

**TABLE 6:** Hemodynamic concentrations in the Simon task in all channels.

		Control group (μ mol)		HIIT group (μ mol)		Tabata group (μ mol)	
Channels	Condition	Pre	Post	Pre	Post	Pre	Post
4	Congruent	0.022 ± 0.05	0.009 ± 0.02	−0.025 ± 0.04	0.033 ± 0.04	0.007 ± 0.04	0.009 ± 0.04
	Incongruent	−0.016 ± 0.05	0.024 ± 0.02	0.013 ± 0.02	−0.011 ± 0.04	0.007 ± 0.04	0.009 ± 0.04
5	Congruent	**0.017 ± 0.06**	**−0.025 ± 0.03**	**−0.018 ± 0.07**	**0.028 ± 0.05**	**−0.026 ± 0.05**	**0.016 ± 0.04**
	Incongruent	−0.026 ± 0.06	0.021 ± 0.05	−0.015 ± 0.06	−0.016 ± 0.04	−0.026 ± 0.05	0.016 ± 0.04
6	Congruent	0.022 ± 0.04	0.001 ± 0.03	0.022 ± 0.08	0.019 ± 0.04	−0.016 ± 0.05	0.004 ± 0.03
	Incongruent	−0.016 ± 0.05	0.020 ± 0.02	−0.015 ± 0.08	0.0002 ± 0.04	−0.016 ± 0.05	0.004 ± 0.03
7	Congruent	0.025 ± 0.07	−0.013 ± 0.05	0.002 ± 0.03	0.014 ± 0.04	−0.023 ± 0.04	0.008 ± 0.04
	Incongruent	−0.003 ± 0.05	0.020 ± 0.03	−0.001 ± 0.03	0.008 ± 0.05	−0.023 ± 0.04	0.008 ± 0.04
8	Congruent	0.010 ± 0.04	−0.011 ± 0.04	−0.003 ± 0.02	0.019 ± 0.04	−0.014 ± 0.03	0.002 ± 0.03
	Incongruent	−0.010 ± 0.03	0.034 ± 0.05	0.001 ± 0.02	−0.003 ± 0.04	−0.014 ± 0.03	0.002 ± 0.03
9	Congruent	−0.052 ± 0.14	0.004 ± 0.02	−0.003 ± 0.05	−0.030 ± 0.12	−0.004 ± 0.03	0.003 ± 0.05
	Incongruent	0.112 ± 0.27	0.011 ± 0.02	−0.008 ± 0.05	−0.020 ± 0.10	−0.004 ± 0.03	0.003 ± 0.05
10	Congruent	0.015 ± 0.05	0.002 ± 0.03	0.004 ± 0.02	0.016 ± 0.05	−0.013 ± 0.03	0.013 ± 0.03
	Incongruent	−0.008 ± 0.04	0.010 ± 0.03	−0.004 ± 0.03	−0.001 ± 0.04	−0.013 ± 0.03	0.013 ± 0.03
11	Congruent	0.008 ± 0.05	0.015 ± 0.03	−0.007 ± 0.03	0.021 ± 0.04	−0.022 ± 0.04	0.013 ± 0.04
	Incongruent	0.036 ± 0.10	0.012 ± 0.03	0.0004 ± 0.03	−0.006 ± 0.04	−0.022 ± 0.04	0.013 ± 0.04
12	Congruent	0.016 ± 0.05	−0.005 ± 0.03	−0.001 ± 0.02	0.026 ± 0.05	−0.012 ± 0.03	0.012 ± 0.04
	Incongruent	0.037 ± 0.12	0.007 ± 0.01	−0.007 ± 0.03	−0.008 ± 0.04	−0.012 ± 0.03	0.012 ± 0.04
13	Congruent	0.019 ± 0.04	0.002 ± 0.03	−0.002 ± 0.02	0.026 ± 0.04	−0.023 ± 0.03	0.008 ± 0.03
	Incongruent	0.007 ± 0.07	0.010 ± 0.02	0.002 ± 0.03	−0.006 ± 0.03	−0.023 ± 0.03	0.008 ± 0.03
14	Congruent	0.022 ± 0.04	0.012 ± 0.04	−0.010 ± 0.04	0.021 ± 0.04	0.002 ± 0.05	0.015 ± 0.03
	Incongruent	−0.002 ± 0.05	0.010 ± 0.03	0.004 ± 0.04	0.002 ± 0.04	0.002 ± 0.05	0.015 ± 0.03
15	Congruent	−0.005 ± 0.05	0.014 ± 0.02	−0.014 ± 0.03	0.030 ± 0.05	−0.005 ± 0.03	0.016 ± 0.06
	Incongruent	0.080 ± 0.21	0.001 ± 0.02	0.003 ± 0.04	−0.004 ± 0.06	−0.005 ± 0.03	0.016 ± 0.06
16	Congruent	0.001 ± 0.09	0.009 ± 0.03	−0.008 ± 0.04	0.009 ± 0.06	−0.014 ± 0.04	0.017 ± 0.05
	Incongruent	0.018 ± 0.07	0.015 ± 0.01	−0.0003 ± 0.03	−0.011 ± 0.05	−0.014 ± 0.04	0.017 ± 0.05
17	Congruent	0.025 ± 0.03	0.004 ± 0.04	0.002 ± 0.04	0.019 ± 0.04	−0.025 ± 0.05	0.006 ± 0.05
	Incongruent	−0.004 ± 0.04	0.018 ± 0.02	−0.006 ± 0.03	−0.0004 ± 0.04	−0.025 ± 0.05	0.006 ± 0.05
18	Congruent	0.019 ± 0.04	0.017 ± 0.03	−0.010 ± 0.05	0.017 ± 0.04	−0.019 ± 0.04	0.003 ± 0.04
	Incongruent	−0.016 ± 0.03	0.014 ± 0.03	0.006 ± 0.04	−0.003 ± 0.03	−0.019 ± 0.04	0.003 ± 0.04
19	Congruent	0.010 ± 0.04	0.013 ± 0.03	0.004 ± 0.05	0.022 ± 0.04	−0.025 ± 0.07	0.002 ± 0.04
	Incongruent	0.006 ± 0.05	0.014 ± 0.03	0.001 ± 0.04	−0.005 ± 0.04	−0.025 ± 0.07	0.002 ± 0.04
20	Congruent	0.019 ± 0.05	−0.019 ± 0.03	0.006 ± 0.04	0.012 ± 0.08	−0.021 ± 0.04	0.007 ± 0.07
	Incongruent	−0.017 ± 0.06	0.023 ± 0.03	−0.022 ± 0.04	0.013 ± 0.07	−0.021 ± 0.04	0.007 ± 0.07
21	Congruent	0.018 ± 0.04	0.002 ± 0.04	0.005 ± 0.03	0.023 ± 0.05	−0.005 ± 0.03	0.009 ± 0.03
	Incongruent	−0.006 ± 0.04	0.009 ± 0.04	−0.005 ± 0.03	−0.004 ± 0.04	−0.005 ± 0.03	0.009 ± 0.03

Oxy-Hb concentrations (μmol) from pre- to post-test in the control, HIIT, and Tabata groups are presented. Data are presented as the mean ± SD. Bold values indicate a significant condition × time × group interaction (*p* < 0.05).

## Discussion

In this study, we aimed to examine the activation of the PFC in response to an acute HIIT or Tabata intervention during inhibitory control testing in healthy young adults. Inhibitory control performance was evaluated by two tasks: the Stroop task and Simon task, and task-related cortical activation was assessed in the form of oxygenated hemoglobin by fNIRS. To our knowledge, this study presents the first experimental evidence for the effects of an acute bout of equipment-free, whole-body Tabata training on inhibitory control and cortical activation in young adults.

### Changes of inhibitory control after HIIT and Tabata training

Regarding the behavioral measurements, inconsistent with our hypothesis, the behavioral results of the presented study demonstrated that there was no significant condition × time × group interaction for RT and ACC in both the Stroop task and Simon task. These results suggested that compared with the control condition, HIIT, and Tabata training did not enhance the inhibitory control performance of the Stroop task and Simon task in young adults. This is inconsistent with previous studies that assessed effects of an acute bout of high-intensity exercise on inhibitory control performance in adults (Kujach et al., 2018; Khandekar et al., 2022). Both Kujach et al. (2018) and Khandekar et al. (2022) found exercise improved inhibitory control.

In contrast, Sudo et al. (2017) reported no significant differences in young adults’ executive function after exhaustive exercise. Hyodo et al. (2017) found that neither slow aerobic dance nor cycling influenced Stroop performance in older adults.

In our study, no condition by time by group interactions were detected in task performance, perhaps due to the arousal hypothesis, which has provided an explanation for the inverted U-shaped function of task performance against physical strength (Yerkes and Dodson, 1908). As arousal states increase with physical exertion, cognitive performance improves to an optimal point, after which further enhancement in physical exertion leads to decreased arousal levels and impaired cognitive performance (Tomporowski and Ellis, 1985). Therefore, researchers have proposed that moderate-intensity exercise brings the arousal state to the optimal point for cognitive function whereas weak and strong exercises do not (Chmura et al., 1994), and when the physical load becomes too heavy, an acute bout of exercise possibly points to impairments in cognitive function (Labelle et al., 2013). The physical exertion required of participants in the current study was set at 80% MAP (HIIT group) and 80–95% of HR max (Tabata group), which may correspond to the decline phase of the inverted U-shaped function. Thus, it is plausible that an acute bout of HIIT and Tabata training caused a decreased arousal state and further induced attenuated benefits for inhibitory control performance in the Stroop task and Simon task.

### Changes in PFC cortical activation after HIIT and Tabata training

We hypothesized that both acute HIIT and Tabata training would stimulate PFC cortical activation in young adults. From baseline to post-test, the participants in the HIIT group showed increased activation during the congruent aspect of the Stroop task and decreased activation during the incongruent aspect of the Stroop task; participants in the Tabata group showed increased activation during the congruent aspect of the Simon task compared to individuals in the control group. These results might indicate that functional neuroimaging is more sensitive than performance measures to acute exercise-induced changes, despite similar task performance.

In the congruent condition of the Stroop task, the HIIT participants elicited greater activation over time in the DLPFC and VLPFC than individuals in the control group. This result was similar to the finding of Kujach et al. (2018) that acute HIIT stimulated cortical activation in the DLPFC during inhibitory control tasks in young participants. Khandekar et al. (2022) found mixed results. They reported a decrease in activation in R-DLPFC, L-VLPFC, and L-FPA after HIIT. They also found an increase in activation in L-DLPFC which was similar to our findings.

Regarding the incongruent condition of the Stroop task, intriguingly, the HIIT group showed decreased activation over time compared to the control group. This result is consistent with the finding of Khandekar et al. (2022) of a decrease in activation in R-DLPFC, L-VLPFC, and L-FPA after HIIT, while it is contrary to the finding of Kujach et al. (2018) that reported that acute HIIT stimulated cortical activation in DLPFC.

One possible reason for the decreased activation that appeared in the HIIT group during the incongruent aspect of the Stroop task in the current study was that the adults became familiar with the Stroop task and were able to perform it more efficiently in their second visit. Another reason could be the time window (48 h) between the first visit and the second visit was too short, so the participants might still have been familiar with the cognitive tasks. The incongruent condition required more inhibitory control abilities than the congruent condition in the Stroop task, and the HIIT group only exhibited decreased activation over time when performing the incongruent condition trials of the Stroop task. It is plausible that acute HIIT could raise the efficiency in DLPFC and VLPFC regions when more inhibitory control abilities were needed (Krafft et al., 2014). A second reason might be that acute HIIT triggered other parts of the brain regions in addition to the PFC (Banich et al., 2001). In previous studies using fMRI and PET, Stroop attentional conflicts were related of activation to the anterior cingulate cortex (ACC) and DLPFC (Mead et al., 2002; Milham et al., 2003). Nevertheless, we failed to monitor activation in the ACC in the current study because fNIRS cannot capture frontomedian activation due to the limited depth penetration of NIR light (Villringer and Chance, 1997).

In the congruent condition of the Simon task, the Tabata group showed enhanced activation in the DLPFC, whereas in the incongruent condition of the Simon task, there were no neural activation differences in the Tabata group over time compared to the control group.

One possible reason for the differences in activation patterns and activated brain regions between the HIIT and Tabata groups during the Stroop task and Simon task might be different cognitive demands in the two tasks. Although both tasks are logically similar and are widely acknowledged to assess inhibitory control, the cognitive demands might be different between these two tasks. The Simon effect emerges because responses are faster and more accurate when the stimulus position and response position correspond (e.g., right stimulus-right response) than when they do not (e.g., right stimulus-left response). The Simon effect results from the automatic coding of stimulus position, which, in turn, automatically activates the spatially corresponding response, thus producing competition between the spatially corresponding response and the response required on the basis of task instructions. The Stroop effect emerges because responses are faster and more accurate when the stimulus meaning and the stimulus color are congruent (e.g., the word “Red” printed in red) rather than incongruent (e.g., the word “Red” printed in green). The Stroop effect results from the automatic coding of the name of stimulus; thus, it also produces a competition between the meaning corresponding response and the response required on the task instructions, which is different from the competition produced by the Simon effect (Pratte et al., 2010; Scerrati et al., 2017). A second reason might be the different exercise modalities of HIIT and Tabata training. Zhu et al. (2021) compared executive function performance under two exercise modalities (i.e., running vs. cycling) of the same intensity; they found that executive function was better immediately after high-intensity cycling but not high-intensity running and was better in 10 min after moderate-intensity cycling but not moderate-intensity running. This suggested that running and cycling might affect executive function differently, although they were designed to be the same intensity. The exercise modalities were different in our HIIT and Tabata groups (cycling and bare-handed, respectively); therefore, it is also possible that their different exercise modalities might differentially affect the brain activation patterns during inhibitory control tasks. Future studies can explore the neurophysiological mechanisms by which exercise modalities differentially affect executive function and related brain activation.

Acute exercise-elicited brain activation alterations in the current study appeared in the DLPFC and VLPFC regions, and both of these regions play an important role in inhibitory control (Jones and Graff-Radford, 2021). The DLPFC is involved in the top-down processing of behavioral responses and decision making (Heekeren et al., 2004); specifically, it is linked to monitoring and processing cognitive conflict in a Stroop paradigm (Banich et al., 2001; Mead et al., 2002; Milham et al., 2003). The VLPFC has been shown to be modulated by manipulating the probability of non-targets, thereby affecting response-inhibition demands (Casey et al., 2001), and animal lesion research has also implicated VLPFC regions in response inhibition (Iversen and Mishkin, 1970; Butters et al., 1973). Therefore, it could be concluded that the acute HIIT and Tabata training in our study leads to altered cortical activation in the brain regions that play a critical role in facilitating inhibitory control processing.

### Changes in cortical activation after HIIT and Tabata training in the right hemisphere

Our results showed that acute exercise elicited brain activation alterations in the right hemisphere, and these results were inconsistent with studies that revealed acute exercise-induced left-lateralized cortical activation in adults (Yanagisawa et al., 2010; Byun et al., 2014; Hyodo et al., 2016; Kujach et al., 2018). In contrast, several studies have reported exercise-induced contralateralized cortical activation, similar to our results. Hyodo et al. (2012) investigated right-hemisphere cortical activation in the FPA after acute moderate-intensity exercise, which is thought to compensate for neural dysfunction specific to task demands in older adults. However, the participants recruited in our study were all healthy young adults without any neurological or psychiatric disorders; thus, it is unlikely that activation in the contralateral hemisphere serves as a compensatory function for our young adult participants. Garavan et al. (1999) indicated a right-hemisphere dominance in response to inhibitory control, and this right lateralization is also consistent with several neuroimaging studies in normal participants (Kawashima et al., 1996; Humberstone et al., 1997; Konishi et al., 1998). However, this study did not involve an acute exercise intervention. Thus, why acute exercise induces inhibitory control to be lateralized to the right hemisphere is uncertain.

### Neurobiological mechanism behind changes of cortical activation after HIIT and Tabata training

It has remained unclear what physiological changes in the brain cause exercise-induced neural activation. However, it seems widely agreed that exercise induces the release of various neurotransmitters from several neuromodulatory systems, including ascending projections to the PFC (Dietrich and Audiffren, 2011). Previous animal studies have indicated that an acute bout of exercise stimulated the release of acetylcholine from the nucleus basalis of Meynert (Kurosawa et al., 1993) and the release of dopamine in the nucleus accumbens (Meeusen and De Meirleir, 1995); moreover, an acute bout of exercise also enhanced the basal levels of noradrenaline in the locus coeruleus (Dishman et al., 1997). In addition, the catecholamine hypothesis demonstrated that acute exercise stimulated monoamine systems and enhanced the release of neurotransmitters such as noradrenaline and dopamine into brain regions, which provided a plausible explanation for the improvement of the cognitive process after acute exercise in adults (McMorris et al., 2008; McMorris, 2016, 2021). Therefore, a possible neurobiological mechanism is that acute HIIT and Tabata training evoke monoamine systems and in turn stimulate prefrontal cortical activation associated with inhibitory control. However, our HIIT intervention was repeated with the same movement, which was extremely tiring. Compared with the Tabata group, the HIIT group might have felt less pleasure from the exercise, causing negative emotions.

Some researchers reported that HIIT enhanced neurotrophic factor levels in animals. Hugues et al. (2022) showed that HIIT upregulated insulin-like growth factor-1 (IGF-1) as well as neurotrophic, metabolic, and angiogenic markers in rats. Abbasian and Asghar Ravasi (2020) found that three weeks of HIIT led to more expression and delivery of brain-derived neurotrophic factor (BDNF) in the brain and plasma through the PGC-1α-pathway. Constans et al. (2021) reported that levels of key brain plasticity markers increased in the hippocampus after 8 weeks of HIIT, though it did not improve cognitive functions. Therefore, it is also possible that exercise-induced neural activation is associated with increasing neurotrophic factors levels.

### Limitations

This study has several limitations. First, despite task familiarization prior to the start of the experiment, practice effects in all groups may have occurred. One explanation for this might be successful strategy use and a less demanding stimulus-response relation due to more automatic motor responses, that is, the process of rule learning over time (Boettiger and D’Esposito, 2005). The option to decrease the influence of practice effects on task performance is probably increasing the task demands, including modifying the task version. However, we abstained from raising the degree of difficulty of the Stroop task and Simon task in the participants’ second visit, which may be one of our limitations. Second, although exercise-induced arousal levels might improve young adults’ inhibitory control, we did not measure the arousal levels by using the Two-Dimensional Mood Scale (TDMS). Third, as mentioned before, due to the limitation of fNIRS measurement, we failed to detect signals of deep brain regions, such as the ACC. Fourth, this study was only conducted with young adults; therefore, the findings cannot be directly generalized to other age groups. Future studies are needed to verify the findings in broader age groups. Fifth, the time (48 h) between the first visit and the second visit was too short, so participants might still have been familiar with the cognitive tasks.

## Conclusion

The present study reveals that from baseline to post-test, an acute bout of HIIT and Tabata training does not lead to beneficial effects on inhibitory control performance in young adults. Despite the absence of task performance differences, our study provides strong new empirical evidence that acute HIIT and Tabata cause alterations in cortical activation related to inhibitory control in young adults. Specifically, participants assigned to the HIIT group demonstrated increased activation during the congruent aspect of the Stroop task and decreased activation during the incongruent aspect of the Stroop task, and the Tabata group showed increased activation during the congruent aspect of the Simon task compared to the control group, suggesting that functional neuroimaging may be more sensitive than performance measures to acute exercise-induced changes. Future studies can explore the underlying mechanisms of different patterns of exercise-induced changes in brain activation for the two tasks.

## Data availability statement

The raw data supporting the conclusions of this article will be made available by the authors, without undue reservation.

## Ethics statement

The studies involving humans were approved by the Ethical Committee of the Medical School, Shenzhen University, Shenzhen, China. The studies were conducted in accordance with the local legislation and institutional requirements. The participants provided their written informed consent to participate in this study.

## Author contributions

XS: investigation, conceptualization, methodology, experimental design, writing—review and editing, supervision, project administration, and funding acquisition. LH: experimental design, data collection, data analysis, visualization, writing—original draft, and writing—review and editing. YL and YF: experimental design, data collection, data analysis, and visualization. All authors had read and approved the submitted manuscript.
